# Supplementary material for: A Reliable Approach for Revealing Molecular Targets in Secondary Ion Mass Spectrometry
Source: Int J Mol Sci. 2022 Apr 21;23(9):4615. doi: 10.3390/ijms23094615 (PMC9103194; doi:10.3390/ijms23094615)
Supplement: Supplementary file 1 [file ijms-23-04615-s001.zip › ijms-1643966-supplementary.pdf]

A reliable approach for revealing molecular targets in secondary ion mass spectrometry

Fengxia Li [1,2,*], Eugenio F. Fornasiero [1,2], Tal M. Dankovich [1,3], Verena Kluever [1], and Silvio O. Rizzoli [1,2,*]

[1] Center for Biostructural Imaging of Neurodegeneration, University Medical Center Göttingen, von-Siebold-Straße 3a, 37075 Göttingen, Germany
[2] Department of Neuro- and Sensory Physiology, University Medical Center Göttingen, Humboldtallee 23, 37073 Göttingen, Germany
[3] International Max Planck Research School for Neuroscience, Göttingen, Germany

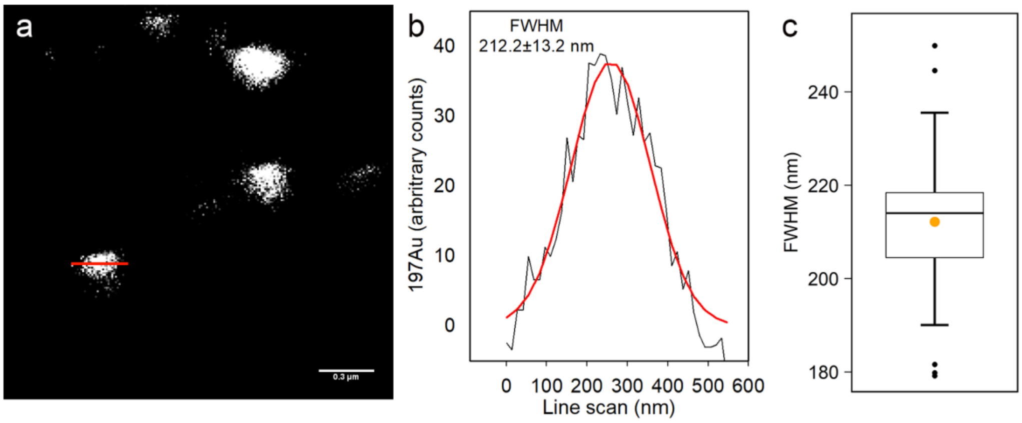

Figure S1. Au standard imaged in nanoSIMS. (a) A representative image of gold spots measured by nanoSIMS; (b) Line scan and fitted Gaussian curve of the gold counts from the red line indicated in (a); (c) Boxplot of FWHW based spatial resolution calculated for multiple detected gold spots (N = 64).

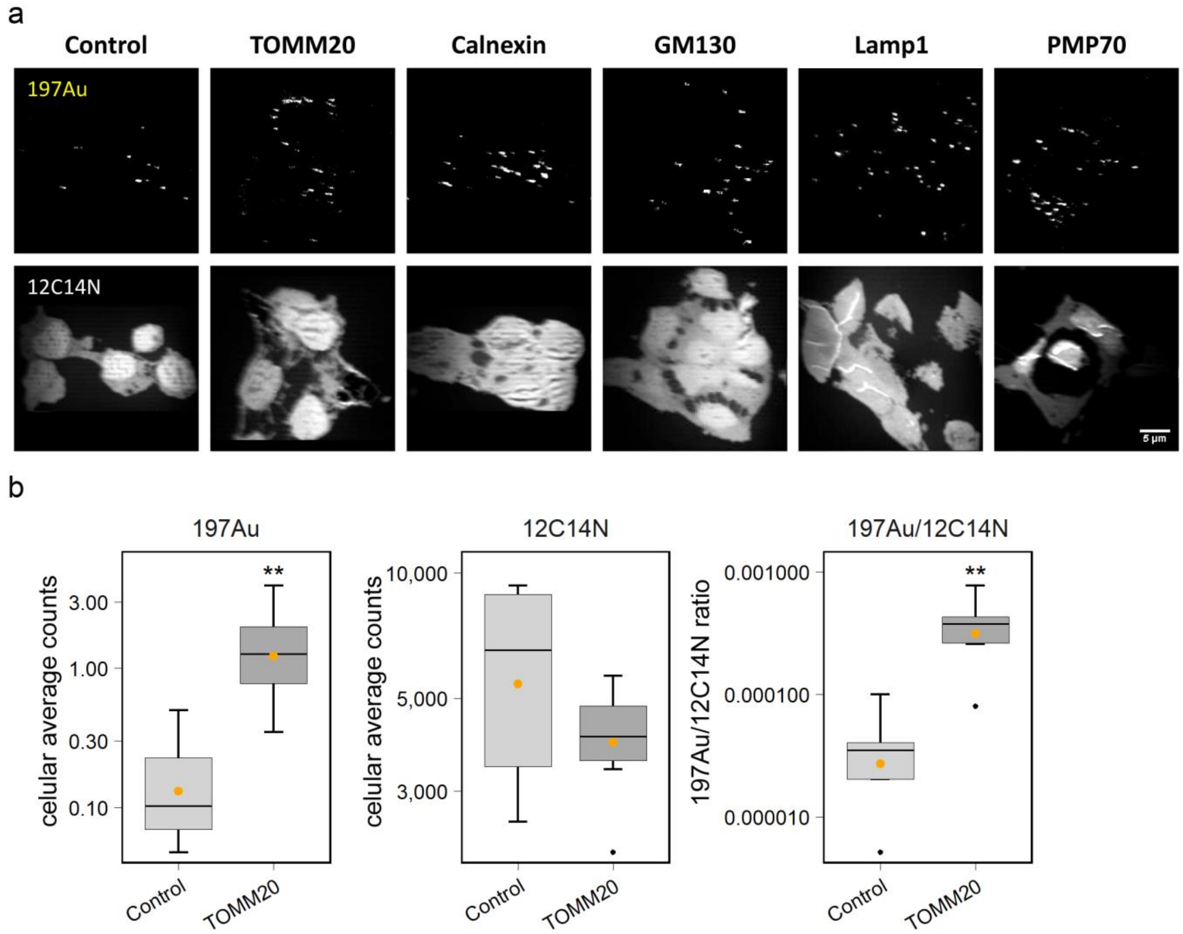

Figure S2. NanoSIMS measurement of 15nm Au_Np labelled HEK293 organelle markers. (a) Representative images of each organelle marker labelling and control staining without primary antibody application; (b) Labelling intensity comparison for TOMM20 stained with 15 nm Au_Np. Wilcoxon rank sum test; N=5 and 7 for control and TOMM20, respectively; p=0.0051, 0.34 and 0.0051 for 197Au, 12C14N and 197Au/12C14N, respectively.

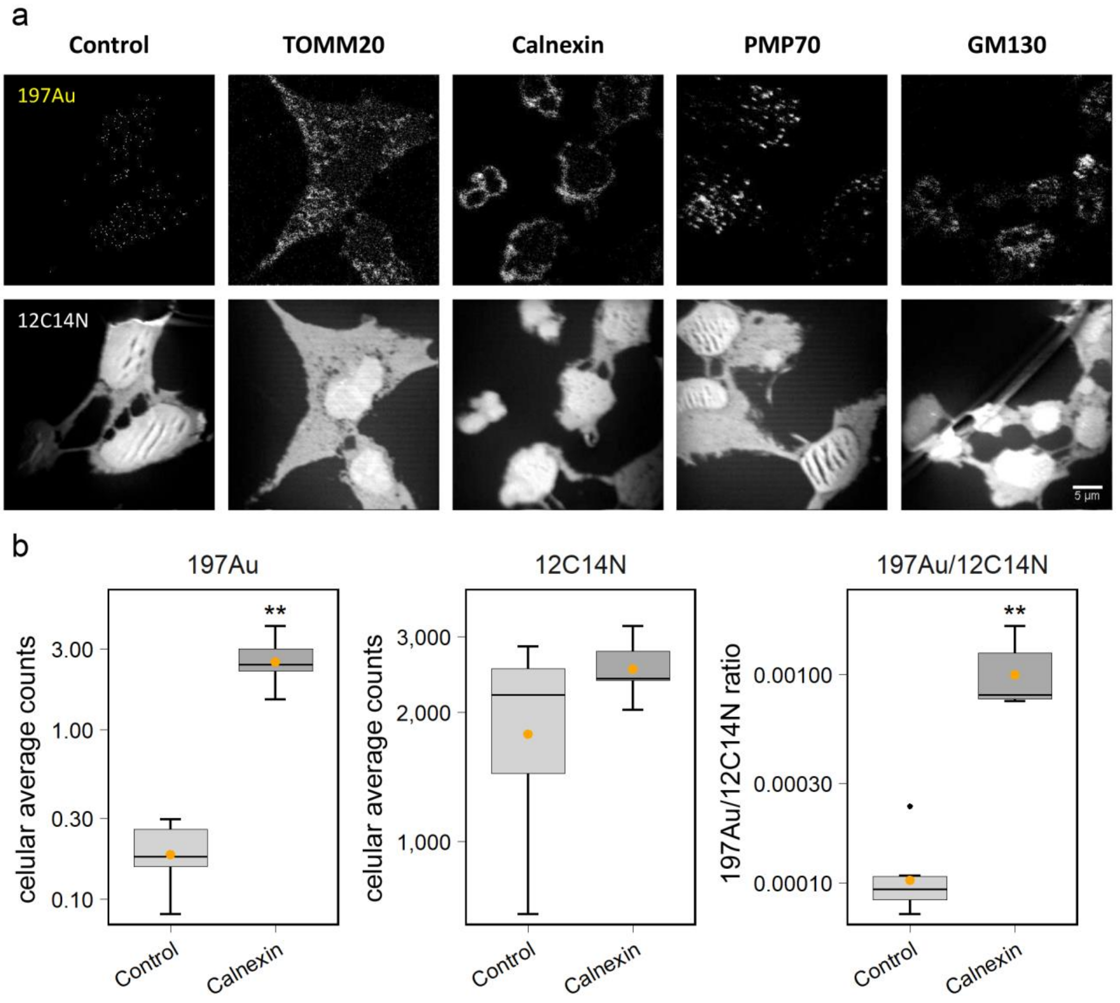

Figure S3. NanoSIMS measurement of 1.4nm Au_Np labelled HEK293 organelle markers. (a) Representative images of each organelle marker labelling and control staining without primary antibody application; (b) Labelling intensity comparison for calnexin staining stained with 1.4 nm Au_Np. Wilcoxon rank sum test; N=7 and 5 for control and calnexin, respectively; p=0.0025, 0.2 and 0.0025 for 197Au, 12C14N and 197Au/12C14N, respectively.

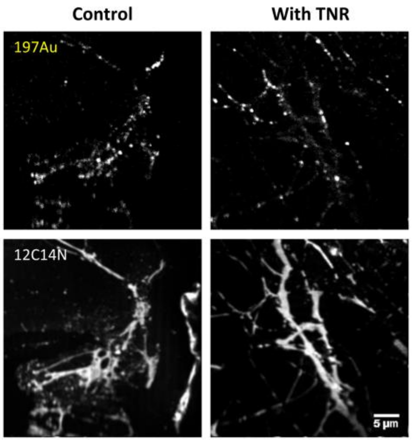

Figure S4. NanoSIMS measurement of 5 nm Ni-NTA-Nanogold lively labelled neuronal cultures detected strong gold signal both in control samples with only gold probe applied and in samples with mixture of His-tagged TNR and gold probe applied.

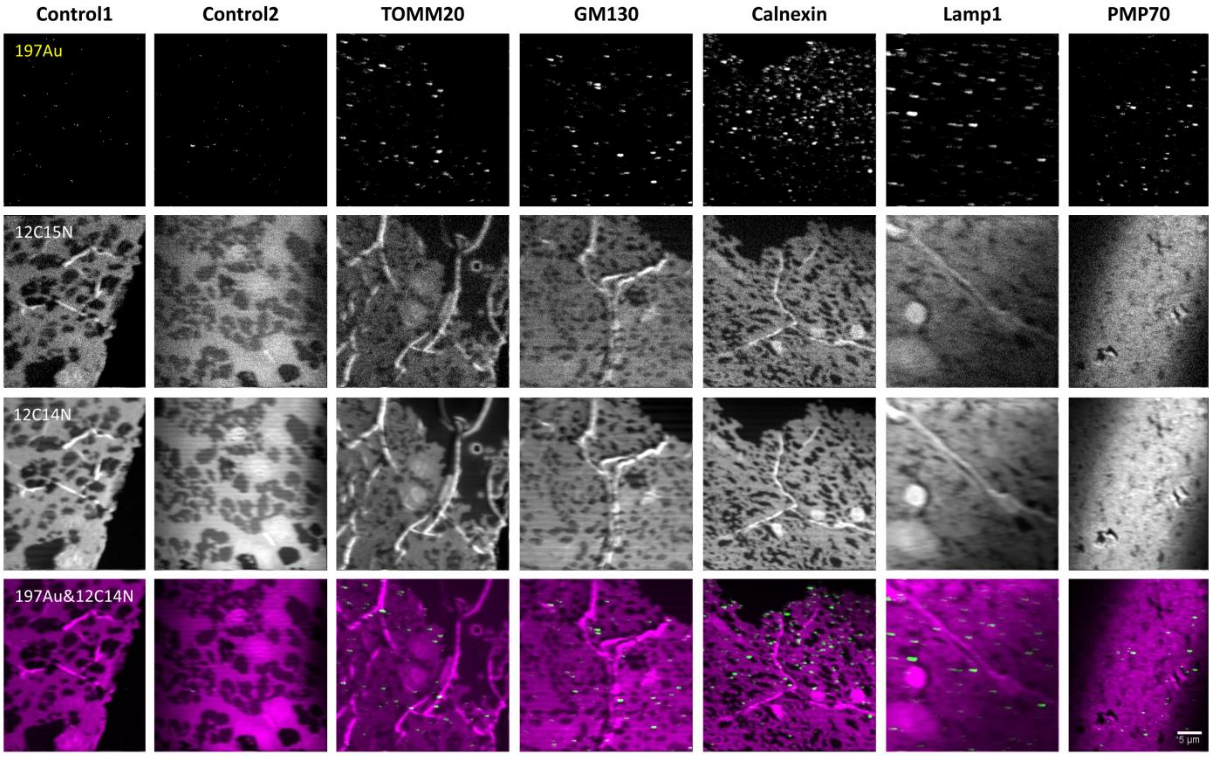

Figure S5. NanoSIMS detection of organelle markers in mouse brain slices tagged by 6 nm Au_Np. 12C15N and 12C14N were imaged simultaneously in a single measurement to allow accurate ratio calculation. 197Au (green) and 12C14N (purple) are merged in the lowest panel.
